# The Key Roles of ROS and RNS as a Signaling Molecule in Plant–Microbe Interactions

**DOI:** 10.3390/antiox12020268

**Published:** 2023-01-25

**Authors:** Murtaza Khan, Sajid Ali, Tiba Nazar Ibrahim Al Azzawi, Saddam Saqib, Fazal Ullah, Asma Ayaz, Wajid Zaman

**Affiliations:** 1 Department of Horticulture and Life Science, Yeungnam University, Gyeongsan 38541, Republic of Korea; 2 Department of Applied Biosciences, Kyungpook National University, Daegu 41566, Republic of Korea; 3 State Key Laboratory of Systematic and Evolutionary Botany, Institute of Botany, Chinese Academy of Sciences, Beijing 100093, China; 4 University of Chinese Academy of Sciences, Beijing 100049, China; 5 State Key Laboratory of Grassland Agro-Ecosystems, School of Life Sciences, Lanzhou University, Lanzhou 730000, China; 6 State Key Laboratory of Biocatalysis and Enzyme Engineering, School of Life Sciences, Hubei University, Wuhan 430062, China; 7 Department of Life Sciences, Yeungnam University, Gyeongsan 38541, Republic of Korea

**Keywords:** ROS, RNS, signaling, plant–microbe interactions, antioxidant system

## Abstract

Reactive oxygen species (ROS) and reactive nitrogen species (RNS) play a pivotal role in the dynamic cell signaling systems in plants, even under biotic and abiotic stress conditions. Over the past two decades, various studies have endorsed the notion that these molecules can act as intracellular and intercellular signaling molecules at a very low concentration to control plant growth and development, symbiotic association, and defense mechanisms in response to biotic and abiotic stress conditions. However, the upsurge of ROS and RNS under stressful conditions can lead to cell damage, retarded growth, and delayed development of plants. As signaling molecules, ROS and RNS have gained great attention from plant scientists and have been studied under different developmental stages of plants. However, the role of RNS and RNS signaling in plant–microbe interactions is still unknown. Different organelles of plant cells contain the enzymes necessary for the formation of ROS and RNS as well as their scavengers, and the spatial and temporal positions of these enzymes determine the signaling pathways. In the present review, we aimed to report the production of ROS and RNS, their role as signaling molecules during plant–microbe interactions, and the antioxidant system as a balancing system in the synthesis and elimination of these species.

## 1. Introduction

Due to their immobile nature, plants are constantly exposed to biotic and abiotic stressors [1,2]. In response to these stressors, different signaling molecules are produced in plants, including melatonin, ROS, and RNS [3,4]. ROS and RNS are key signaling molecules and play a pivotal role in the regulation of different processes, including plant growth and development, metabolism, and the response to stressful (biotic and abiotic) conditions [5,6]. In plants, ROS are produced in a variety of cellular compartments, including chloroplasts, mitochondria, and peroxisomes [7]. In addition to causing irreversible DNA damage and cell death, ROS are significant signaling molecules that control healthy plant growth and responses to stress [7]. This shows that ROS have a dual role in vivo with various levels of reactivity, generation locations, and capabilities to penetrate biological membranes. RNS in plants were first identified in the 1960s, but until the 1990s, they did not receive the same level of attention as their oxygen equivalents (ROS). Among RNS, nitric oxide (NO), acting as a signaling molecule in plant disease, rekindled interest among researchers to understand the physiological roles of RNS in plants [8]. RNS were initially recognized through their signaling role, in contrast to ROS, which were first recognized as harmful chemicals and then signals [5]. The importance of ROS and RNS as signals and significant regulators of a number of activities in plants, such as metabolism, growth and development, and response to biotic and abiotic stressors, is now well documented [6]. Various biotic and abiotic stress factors, including intense light, extreme temperature, salt, drought, waterlogging, and plant pathogens, cause the induction of ROS and RNS in plants [9,10,11].

Broadly, ROS are divided into two classes: radicals and non-radicals [12]. Radical ROS include superoxide anions (O_2_^−^), hydroxyl radicals (^−^OH), alkoxyl (RO^−^), hydroperoxyl (HO_2_^−^), peroxyl (ROO^−^), carbonate (CO_3_^−^), and semiquinone (SQ^−^). Non-radical ROS include hypobromous acid (HOBr), singlet oxygen (^1^O_2_^−^), ozone (O_3_), hydrogen peroxide (H_2_O_2_), hypoiodous acid (HOI), hypoperoxides (ROOH), and hypochlorous acid (HOCl). It has been established that under both normal and stressful circumstances, ROS can be formed in numerous locations, including the cell wall, mitochondria, chloroplasts, plasma membranes, peroxisomes, and endoplasmic reticulum. When light is present, peroxisomes and chloroplasts are the main sources of ROS production; however, when light is absent, mitochondria are the main source. ROS, which have been identified as the second messenger in intracellular signaling cascades, have been correlated with a range of plant responses, including programmed cell death, stomatal closure, gravitropism, and the development of abiotic and biotic stress tolerance. The activity of numerous signaling molecules, including protein phosphatases, transcription factors, and protein kinases, can also be affected by ROS, in addition to how they interact with other signaling molecules and regulate the response given downstream. The quantity, potency, and size of the ROS signaling pool is all dependent on the equilibrium between the production and the elimination of ROS [13]. 

Similarly, RNS are also divided into two classes: free radicals and non-radicals [14]. Free radical RNS include nitric oxide (NO), nitric dioxide (NO_2_), and nitric oxide (NO_3_). Non-radical RNS include nitrous acid (HNO_2_), nitroxyl anion (NO^−^), nitrosonium cation (NO^+^), peroxynitrite (NOOO^−^), dinitrogen tetroxide (N_2_O_4_), and dinitrogen trioxide (N_2_O_3_). NO, a main source of RNS, is produced in plants through numerous enzymatic and non-enzymatic mechanisms. Because the process of NO generation in plant cells is less understood than that of ROS, it presents one of the biggest difficulties in the research of NO as a signaling molecule. For instance, in animals, NO is produced mostly by the action of nitric oxide synthase (NOS), but in higher plants, NOS has not been discovered yet. However, the proposed pathways for NO production in plants are oxidative and reductive pathways. The substrates for NO production in oxidative pathways are arginine, hydroxylamine, and polyamines, while the substrates for NO production in reductive pathways are xanthine oxidoreductase (XOR), and nitrite:NO-reductase (NiNOR) reductase. RNS are produced in different organelles of plants, including the chloroplast, cell wall, cell membrane, mitochondria, cytoplasm, and peroxisome [15]. Under biotic and abiotic stress situations, plants’ nitric oxide (NO) functions as a signaling molecule in plant growth and development [8,9]. 

Moreover, the recognition of a pathogen leads to “oxidative burst”, which results in ROS and RNS generation and accumulation in higher quantities; by triggering callose deposition, cross-linking of glycoproteins in cell walls, and localized programmed cell death to stop the spread of infection, ROS/RNS can directly affect the pathogen [16]. On the other hand, inoculation with beneficial microbial flora such as plant-growth-promoting rhizobacteria (PGPR) reduces oxidative stress via scavenging overproduced ROS, enhances the antioxidant system of the host plant, and mitigates the adverse effects of stressful conditions [10,17]. 

In this overview, we report the biosynthesis of ROS and RNS, their function as signaling molecules during plant–microbe interactions, and the antioxidant system’s involvement as a balancing mechanism in the synthesis and elimination of these species. The present review covers the research gaps and basic information regarding ROS, RNS generation, and their signaling during plant and microbe interactions.

## 2. Types of ROS

**Figure 1:**
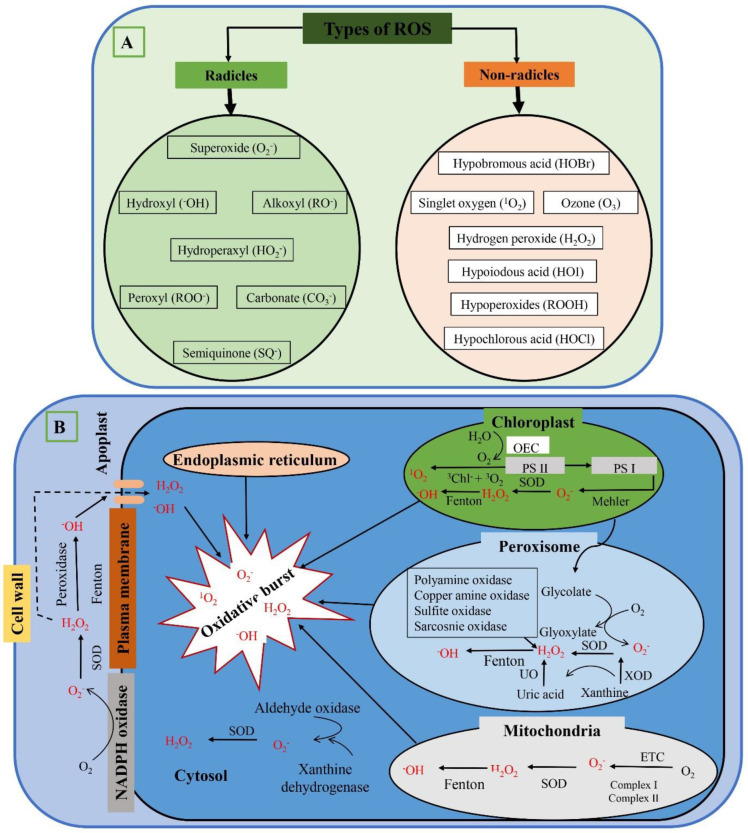
(**A**) All types of reactive oxygen species (ROS) in plants and (**B**) sites of ROS production in the plant cell.

In general, ROS are molecules of oxygen (O_2_) that have undergone incomplete activation or reduction, as well as being the main product or an O_2_-containing molecule byproduct that is more reactive than ambient O_2_. O_2_ molecules’ electrons or energy transfer forms ROS. In plants, ^−^OH, O_2_^−^, ^1^O_2_^−^, and H_2_O_2_ are the most common cellular ROS. ROS are produced by cells in both radical and non-radical forms. Radical ROS include superoxide anions (O_2_^−^), hydroxyl radicals (^−^OH), alkoxyl (RO^−^), hydroperoxyl (HO_2_^−^), peroxyl (ROO^−^), carbonate (CO_3_^−^), and semiquinone (SQ^−^). Non-radical ROS include hypobromous acid (HOBr), singlet oxygen (^1^O_2_^−^), ozone (O_3_), hydrogen peroxide (H_2_O_2_), hypoiodous acid (HOI), hypoperoxides (ROOH), and hypochlorous acid (HOCl). Each form of ROS influences various physiological and biochemical processes in plants that are controlled by various genes and has a distinct oxidative capacity [18]. Different kinds of ROS are shown in Figure 1A, and some of the important ROS are discussed below.

### 2.1. Superoxide Anions (O_2_^−^)

ROS are continuously generated in the chloroplasts as a result of partial O_2_ reduction or energy transfer to O_2_. O_2_^−^ is mostly produced in the thylakoid-localized photosystem I (PSI) and other cellular compartments during the noncyclic electron transport chain (ETC). In a typical reaction between O_2_ and cytochrome c oxidase, H_2_O is produced. O_2_^−^ is occasionally created when O_2_ reacts with the various ETC constituents. Typically, it develops first among ROS. O_2_^−^ may undergo another reaction that could result in the production of more ROS [19]. In an investigation by [20], it was found that O_2_^−^ plays a key role in breaking seed dormancy. These anions, together with ROS, are accountable for the alteration of thiol groups that leads to a decrease in the total glutathione pool, which is crucial for the mobilization of nitrogen and carbohydrate needed for seed germination and development [21]. Furthermore, it has been shown that O_2_^−^ makes antioxidant enzymes such as catalase, peroxidases, and pseudocatalases inactive, leaving cells open to other downstream oxidants, such as H_2_O_2_ [22].

### 2.2. Singlet Oxygen (^1^O_2_^−^)

In contrast to electron transfer to O_2_, this ROS is created by the chlorophyll reaction (in the antenna system, in the triplet state). Stomatal closure is brought on by heavy metals, salt, and dryness, which results in low intracellular CO_2_ concentration. The entire photosynthetic apparatus is put in danger due to significant damage to both photosystems (PSI and PSII) and the facilitated generation of ^1^O_2_^−^, which harms a variety of targets. Furthermore, ROS also harm plant pigments, proteins, lipids, and nucleic acids, which leads to cellular death [23]. In addition to its well-known harmful effects, ^1^O_2_^−^ appears to stimulate various stress responses by activating specific retrograde signaling pathways through the oxidative alteration of carotenoids, proteins, and lipids at sub-lethal levels [24]. 

### 2.3. Hydrogen Peroxide (H_2_O_2_)

Hydrogen peroxide is produced in plant cells both naturally and in response to biotic and abiotic stressors. Both univalent protonation and the reduction of O_2_^−^ result in the production of moderately reactive H_2_O_2_. The main sources of H_2_O_2_ production in plant cells are the endoplasmic reticulum (ER), mitochondria, ETC in the chloroplast, oxidation of fatty acids, photorespiration, and the cell membrane. Low concentrations of H_2_O_2_ are beneficial to plants, but higher concentrations are toxic. At optimum concentration inside the cell, it serves as a regulatory signal for critical physiological processes, such senescence, stomatal movement, photosynthesis and photorespiration, growth, and development [25]. Furthermore, H_2_O_2_ directly kills the microbe at the infection sites, induces cell walls’ lignification, induces phytoalexin’s production, triggers PCD and the HR, and induces SAR [26]. 

### 2.4. Hydroxyl Radical (^−^OH)

The most reactive and harmful ROS is ^−^OH. It is created at neutral pH levels by the Fenton reaction of H_2_O_2_ and O_2_^−^, which is aided by transition metals such as Fe. By inducing lipid peroxidation (LPO), protein degradation, and membrane disintegration, it can affect a number of biological components. The excessive accumulation of ^−^OH leads to cellular death because there is no enzymatic machinery able to remove this harmful radical [27]. Similar to how H_2_O_2_ was formerly thought to be just a harmful byproduct of oxidative metabolism but is now recognized to play signaling roles in plant cells, there is growing evidence that the ^−^OH radical is more than just a destructive agent. Its oxidative capacity aids in germination, growth, stomatal closure, reproduction, the immunological response, and stress adaption [28]. 

### 2.5. Peroxyl Radical (ROO^−^)

The primary chain propagation step in lipid peroxidation and non-lipid systems is the creation of RO2- and RO- radicals, which can be triggered by the heating-induced breakdown of protein and lipid peroxides or by the addition of transition-metal ions. The oxidation of lipids, DNA damage, modifications to the structure of proteins, and food breakdown are all significantly influenced by the peroxyl radical [27].

### 2.6. Alkoxy Radical (RO^−^) 

Alkoxyl radicals are produced during lipid oxidative degradation or peroxidization without the aid of enzymes through the Fenton reaction, electron reductions, or the fusion of two peroxyl radicals. Alkoxyl radicals can damage DNA and trigger apoptosis since they are potent oxidizers [27]. 

## 3. ROS Production Locations

It has been demonstrated that ROS can be created both normally and under stressful circumstances in a variety of locations, including the mitochondria, chloroplasts, plasma membranes, peroxisomes, endoplasmic reticulum, and cell wall. When light is present, ROS are largely created by peroxisomes and chloroplasts; however, when light is absent, ROS are produced by mitochondria. 

### 3.1. Chloroplast

The most prominent site of ROS synthesis is in chloroplasts, where light and chlorophyll (chl) combine to form ROS. In chloroplasts, triplet chl and ETC, including PSI and PSII, create ROS. In the Mehler reaction, in the PSI, SOD changes O_2_^−^ into H_2_O_2_ [29]. When O_2_^−^ and H_2_O_2_ are mixed with metal ions such as Fe^2+^, they produce more highly reactive ^−^OH. Numerous environmental stresses promote stomatal closure, which lowers CO_2_ levels and leads to the production of chloroplastic ROS [30]. 

### 3.2. Mitochondria

Mitochondria are also in charge of producing harmful ROS, such as O_2_^−^ and H_2_O_2_, but on a lower scale. This is caused by the mitochondrial ETC’s (mt ETC) abundance of energetic electrons, which can decrease O_2_ and generate ROS. Complexes I and III are the two main mt ETC elements in charge of producing ROS [31]. In addition, the mitochondrial matrix is home to a large number of ROS-producing enzymes. In mitochondria, Mn-SOD and APX reduce O_2_ into H_2_O_2_. ROS generation increases noticeably when mitochondria are under abiotic stress [32]. 

### 3.3. Apoplast

Incoming CO_2_ appears to be transformed into a soluble, diffusible form that may be carried into the cytoplasm to carry out photosynthesis via the diffusible region surrounding the plant cell membrane. Stress signals and abscisic acid work together to make the apoplast a prominent site for H_2_O_2_ generation under adverse environmental conditions. Other compounds that produce ROS include cell-wall-linked oxidases, polyamine oxidases, and pH-dependent peroxidases (POXs) [33]. 

### 3.4. Plasma Membranes

A plasma membrane encircles each plant cell, continually interacting with the environment to provide vital information for the cells’ survival. Dismutase is either spontaneously transformed into H_2_O_2_ or is catalyzed by NADPH oxidase during the transport of electrons from cytosolic NADPH to O_2_, and SOD creates O_2_^−^. It is widely known that NADPH oxidase plays a crucial role in plants’ defense mechanisms against pathogenic infection and abiotic stress [34]. 

### 3.5. Cell wall and Endoplasmic Reticulum

Polyunsaturated fatty acid (PUFA) hydroperoxidation is produced by the cell-wall-localized lipoxygenase (LOX), making it an active producer of reactive oxygen species (ROS) such as O_2_^−^, ^−^OH, ^1^O_2_, and H_2_O_2_. Using diamines or polyamines, cell-wall-localized diamine oxidases produce ROS in the cell wall. Recombinant lignin is produced as a result of the extensive cross-linking that occurs during the pathogenic attack on lignin precursors via H_2_O_2_-mediated pathways [35]. Through NADPH-mediated electron transport, Cyt P450, which is found in the ER, produces O_2_^−^. 

The total amount of ROS produced is the sum of the ROS produced in these cellular organelles. The overproduction of ROS is known as oxidative burst, and it may cause oxidative stress. The cites of ROS production are shown in Figure 1B.

## 4. ROS Plays a Significant Role in Plant–Microbe Interactions

The development of abiotic and biotic stress tolerance in plants, programmed cell death, stomatal closure, gravitropism, and other responses have all been correlated with ROS, which have been identified as the second messengers in intracellular signaling cascades [36]. Additionally, ROS can interact with protein phosphatases, transcription factors, and protein kinases to alter their activity. The quantity, potency, and size of the ROS signaling pool are all dependent on the equilibrium between oxidant generation and antioxidant clearance [34,35]. 

### 4.1. Role of ROS in PTI

Innate defense mechanisms in plants rely on plasma-membrane-localized pattern recognition receptors (PRRs) to defend against a variety of pathogens [37]. Pattern-triggered immunity (PTI) is the result of PRRs recognizing conserved microbial features known as pathogen-associated molecular patterns (PAMPs) [8]. Through the activation of NADPH oxidases and peroxidases caused by PAMP perception by plants through PRRs, ROS are produced, which in turn induce PTI-dependent basal defenses that thwart encroaching pathogens. Following the perception of PAMP, the produced ROS act as toxic molecules to kill the pathogens [38]. In addition to being signaling molecules, ROS also cause plant immunological and cell death responses [39]. Therefore, pathogens must take precautions to limit their exposure to hazardous ROS. In response to pathogens infection, ROS are produced by the action of NADPH oxidases, also known as respiratory burst oxidase homologs (RBOHs) [39]. The PRR-associated kinase BIK1 has been proposed to be crucial for PAMP-triggered ROS generation via phosphorylating NADPH oxidase (RBOHD) [40]. Additionally, the Arabidopsis PTI is significantly influenced by the apoplastic peroxidase-dependent ROS burst, which is mediated by the detection of PAMPs [41]. The extracellular peroxidase CaPO_2_, found in peppers, also produces bursts of ROS, which activate local and systemic cell death as well as the body’s reaction to bacterial infections [42]. Systemic acquired resistance (SAR) and PTI pathways are activated in response to *Pseudomonas syringae pv. tomato DC3000* and two common PAMPs (flagellin and chitin), respectively, by the plant aquaporin AtPIP;4, which has been shown to cause cytoplasmic import of apoplastic H_2_O_2_ into plant cells [43]. This shows that aquaporins play a crucial part in the signaling of apocytoplastic ROS in disease immune pathways.

Through the fortification of their cell walls, plants protect themselves from invasive infections. An apoplastic H_2_O_2_ burst, cell wall cross-linking, and callose deposition at the site of infection are all factors that aid in cell wall fortifications [44]. For cell maturation and wall toughening during the early phases of plant defense, effector (elicitor)-induced oxidative cross-linking of structural proteins in plant cells is crucial [45]. Callose, a (1,3)-b-glucan, is a key ingredient in the thickening of plant cell walls at the sites of fungal penetration [46]. In order to improve PMR4-dependent callose production and finally confer total penetration resistance to powdery mildew, the Arabidopsis GTPase RabA4c physically interacts with its effector PMR4 [47]. In order to reinforce the cell wall, hydroxyproline-rich glycoproteins (HRGPs) generate intra- and intermolecular cross-links [48]. H_2_O_2_, when exogenously applied to peroxidase knockdown Arabidopsis lines treated with the bacterial flagellin Flg22, restores the callose deposition-deficient phenotype [41]. This shows that PAMP-triggered immunological responses, such as callose deposition, depend on cell wall peroxidase-dependent H_2_O_2_ generation. 

### 4.2. Role of ROS in ETI

Plants have developed a cell-based surveillance system that uses intracellular nucleotide-binding leucine-rich repeat (NLR) receptors to identify certain pathogen effectors, resulting in resistance (R) gene-mediated effector-triggered immunity (ETI). In response to avirulent pathogens, plants overproduce ROS. A powerful ROS burst and the HR cell death response, two essential elements of ETI, are induced as a result of interactions between pathogen avirulence (*Avr*) effectors and NLR proteins [49]. However, uncertainty exists about the activation of HR cell death and immunological responses by NLR-mediated ROS. By preventing the buildup of apoplastic ROS, even in the presence of the R gene, rice resistance to incompatible rice blast fungus isolates is inhibited [50]. Although the Avr effector and cognate NLR proteins remain unaffected, cell death and immunological responses are significantly inhibited in the absence of normal ROS generation [50]. NLR-mediated cell death, immunity, and disease-associated cell death are all dependent on increased ROS generation during infection [51,52]. Although the two stages of plant immunity are spatially and temporally separate, they are closely linked to the burst of ROS. The successful identification of plant pathogens and the activation of plant defenses are both characterized by ROS’s production in plant cells [44]. Furthermore, it is well established that pathogen-induced ROS production in chloroplasts is essential for signaling and/or carrying out hypersensitive response (HR) cell death in plants [44]. H_2_O_2_ activates PTI and systemic acquired resistance (SAR) pathways [44].

### 4.3. Role of ROS in Symbiotic Association

Although the synthesis of ROS is mostly associated with pathogen invasion, their production has also been noticed in other biotic interactions, such as symbiosis between bacteria or mycorrhiza, indicating that ROS synthesis is a common feature of different biotic interactions [39]. The previous results also indicate that after the exposure of plants to beneficial microbes, the synthesis of ROS is low and the level of the antioxidant system is high [53]. Therefore, for ROS to act as signaling molecules, a proper balance between the producers and the scavengers of ROS is crucial, and any disturbance in the equilibrium between them can cause oxidative burst, leading to lipid peroxidation, damage to nucleic acids and proteins, alteration of the metabolism of carbohydrates, and eventually cell death [54].

**Figure 2:**
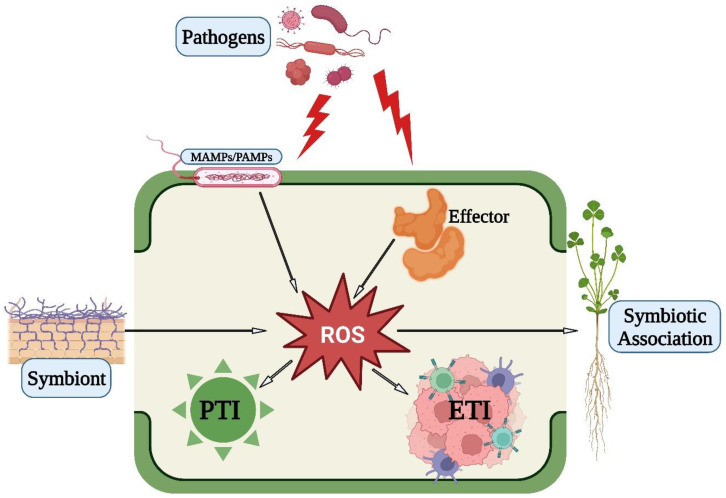
Schematic representation of ROS in PTI, ETI, and symbiotic association.

As a summary, the role of ROS in PTI, ETI, and symbiotic association is shown in Figure 2.

Plants protect themselves from the attack of pathogens and their infection by the activation of hypersensitive response (HR)-mediated cell death, programmed cell death (PCD), lignin biosynthesis, PTI, ETI, expression of defense-related genes, etc. Therefore, both ROS and RNS, as signaling molecules, significantly induce these processes to protect themselves from the attack and infection of pathogens. The overproduction of ROS and RNS, primarily O_2_^−^, H_2_O_2_, and NO, during the oxidative burst may directly contribute to the killing of pathogens, the strengthening of plant cell walls, the induction of the HR, and the production of systemic resistance signaling [8,55]. An essential component of a successful defense mechanism for plants against biotrophic infections feeding on living host tissues has been thought to be the death of assaulted cells during the HR, preceded by an oxidative burst [55,56]. Furthermore, both ROS and NO significantly contribute to the induction of stress-related genes and PCD to protect plants from the adverse effects of the pathogens [55,57]. For instance, in response to different pathogens, ROS induce hypersensitive response (HR)-mediated cell death in tobacco, programmed cell death (PCD) in barley and *Nicotiana benthamiana*, expression of defense-related genes in *N. benthamiana,* lignin biosynthesis and plant immunity in Arabidopsis, and PCD and the HR in tobacco to protect these plants from the infection of pathogenic organisms [58,59,60,61,62,63,64,65,66,67,68,69]. Interestingly, ROS also act as signaling molecules to improve the symbiotic association of beneficial microbes with different plants, including *Medicago truncatula*, *Phaseolus vulgaris*, and *Castanea sativa*, to improve plant growth and development under normal and stressful conditions [69,70,71]. 

Similarly, RNS also act as signaling molecules to induce the defense system of plants to protect them from pathogenic attacks. For instance, in response to different pathogens, RNS induce PTI, ETI, and SAR (different modes of plant defense) in *Arabidopsis*, resistance to viral infection in rice, resistance against tobacco mosaic virus infection in tobacco, and resistance against powdery mildew invasion in wheat [5,8,37,72,73,74]. Interestingly, RNS such as ROS also act as signaling molecules to improve the symbiotic association of beneficial microbes with different plants, including *Medicago truncatula*, *Lotus japonicus*, *Medicago truncatula*, and Legumes [75,76,77,78]. Furthermore, NO, in particular, directs the development of PCD, phytoalexin accumulation, and SA production and signaling in plants [79]. NO differentially regulates the activity of the zinc finger proteins S-Nitrosothiols (SNO) Regulated (SRG) SRG2 and SRG3 in Arabidopsis, which are positive regulators of plant immunity [79]. The roles of ROS and RNS as signaling molecules in plant interaction with harmful and beneficial microbes are given in Table 1.

## 5. Plants Antioxidant Defense System

ROS play a dual role in living organisms, acting as signaling molecules at optimum concentration and toxic molecules at higher concentrations [83]. Therefore, plants have a complex and multilayered network of the antioxidative system (AOS) to protect them from injurious ROS. The cooperation and involvement of AOS in redox processes, which improves safety and promotes regeneration of the active reduced forms, is particularly crucial for the existence of stressed plants [84]. Some of the enzymatic and non-enzymatic antioxidants are discussed below.

### 5.1. Enzymatic Antioxidants

#### 5.1.1. Superoxide Dismutase

SOD (EC 1.15.1.1) significantly reduces oxidative damage by catalyzing the quick dismutation of O_2_^−^ to lower the risk of ^−^OH production in plants. Under stress conditions, SOD eliminates O_2_^−^ and converts it into O_2_ and H_2_O_2_. In a biological system, dismutation reactions occur when oxidation and reduction events occur simultaneously on the same reactant (in this case, O_2_^−^), ultimately resulting in the formation of two compounds: one with a higher oxidation state (such as O_2_) and another with a lower oxidation state (H_2_O_2_ in this case). This enzyme is regarded as one of the primary enzymatic systems responsible for removing free radicals (O_2_^−^) produced by plants under stress [85]. Through a Haber–Weiss reaction, additional enzymes such as CAT and POX cooperate closely with SOD to stop the production of more toxic ROS by both O_2_^−^ and H_2_O_2_. In response to drought and Pb stress, SOD activity was dramatically increased in different cultivars of Phaseolus vulgaris and Oryza sativa [9,86]. In comparison to spontaneous processes, SOD-catalyzed dismutation is 10,000 times faster. SOD is present in all aerobic cells as well as oxidative-stress-sensitive subcellular compartments. Plants have three different varieties of SOD metalloenzymes depending on the type of metal cofactor that is present in the active center. The most prevalent isoenzyme is Cu/Zn-SOD, which is abundant in the chloroplast stroma, cytosol, peroxisomes, and apoplast [84]. Mn-SOD is expressed in mitochondria and peroxisomes, the apoplast, and the cell wall, whereas Fe-SOD is only mildly detectable in these structures. However, only the chloroplast stroma of a few plant species can use this isoenzyme [87].

#### 5.1.2. Catalase and Peroxidase

The most crucial enzymes in the fine regulation of ROS concentration within the cell are catalases (CATs; EC 1.11.1.6) and peroxidases, which control the intracellular amount of H_2_O_2_ in the cell [2]. According to reports, the CAT enzyme exists in a variety of forms and is expressed in various plant tissues at various developmental stages [88]. Given cellular compartmentalization, it is possible that peroxisomes have substantial amounts of CATs, but chloroplasts do not contain this enzyme [84]. The catalases are very efficient in the removal of H_2_O_2_ and split two H_2_O_2_ molecules into water and oxygen. Catalase does not need a reductant to perform its catalytic function because, in this two-step process, H_2_O_2_ first oxidizes the iron in the CAT to produce an intermediate iron peroxide known as compound I. However, at greater H_2_O_2_ concentrations, the second molecule of H_2_O_2_ acts as a reductant for this intermediate chemical I, which results in the regeneration of the enzyme and the release of water and oxygen in the subsequent step [86]. During drought, salinity, and Pb stress, CAT’s activity was significantly enhanced in *Triticum aestivum*, *Cicer arietinum*, and O. sativa. Furthermore, the application of melatonin and sodium nitroprusside (SNP) enhanced the activity of the CAT during drought and under Pb stress [9,89]. 

Peroxidases (POXs; EC 1.11.1.7) are made in the endoplasmic reticulum and Golgi apparatus, where they are then secreted into the vacuoles or extracellular environment. Most plants use POX in its original form, which consists of a single polypeptide with 300–350 amino acid residues and a molecular weight (MW) of 33–55 kDa. Ascorbate and glutathione are the major components in the antioxidant system that regulate redox homeostasis through the Foyer–Halliwell–Asada pathway, while flavonoids, phenolic compounds, and POX act as the second line of defense for plants in dealing with excessive H_2_O_2_ [90,91]. However, POX plays a variety of roles in the growth and development of plants. This family of enzymes is also engaged in cell wall cross-linking, cell wall loosening, lignification, saucerization, and auxin catabolism in addition to its roles in the catabolism of H_2_O_2_ and redox homeostasis. By catalyzing the oxidation of phenolic substrates with H_2_O_2_ acting as an electron acceptor, POX removes the H_2_O_2_. MDA (mono-dehydro-ascorbyl radical), ascorbate, and DHA (de-hydro-ascorbate) were produced as a result of subsequent reactions, as well as the cross-linking product of phenolic compounds such as lignin or suberin. In a recently completed study, POX from *Z. mays* and *P. vulgaris* roots were extracted and found to be functional for cross-linking the globular protein patatin from *Solanum tuberosum*. Such types of enzymatic cross-linking reactions are important for examining protein–protein interactions. It has been discovered that phenolic chemicals facilitate the cross-linking reaction [92]. This study sheds light on the value of POX for comprehending the biophysical structure of the target protein.

Together with the non-enzymatic components of the antioxidant system, the enzymatic components provide a complex and multi-faceted protective mechanism to maintain ROS homeostasis in order to both avoid oxidative-induced damages in plant cells and support plant development. SOD (EC 1.15.1.1) and peroxidases such as CAT (EC 1.11.1.6) and POX (EC 1.11.1.7) constitute the enzymatic antioxidant components. Considering their role as ROS scavengers, variations in their activity and/or transcript accumulation are a common feature in plants under biotic and abiotic stresses [93].

### 5.2. Non-Enzymatic Antioxidants

#### 5.2.1. Ascorbic Acid

The most prevalent AOX metabolite in plant cells is ascorbic acid (AsA), also known as vitamin C. (Smirnoff, 2008). Ascorbate peroxidase’s (APX; EC 1.11.1.11) activity enables it to directly interact with various ROS, neutralize the harmful effects of O_2_^−^, ^−^OH, and ^1^O_2_, and act as an electron donor in enzymatic activities that decrease the amount of H_2_O_2_ [94]. Chloroplasts make up 30–40% of a cell’s overall AsA content and are home to this water-soluble AOX, which can accumulate to a concentration of 300mM in plant cells [95]. However, Castro et al. [96] found that AsA, when at enhanced levels, may operate as a prooxidant in the presence of high H_2_O_2_ levels, activating the Fenton reaction and boosting oxidative stress in rice leaves when exposed to intense UV radiation. In terms of its biosynthesis, L-galactono—lactone dehydrogenase (EC 1.3.2.3)—generates AsA in the mitochondria, where it is later transferred to other organelles by active transport or facilitated diffusion [94]. Under normal circumstances, AsA is mostly found in its reduced form, and its pool is kept constant by the activities of monodehydroascorbate reductase (MDHAR; EC 1.6.5.4) and DHAR [95]. Aside from that, AsA actively contributes to the regulation of mitosis, cellular elongation, senescence, and cell death, in addition to stabilizing enzymes with artificial metallic ions [96].

#### 5.2.2. Glutathione

Tripeptide glutathione (GSH), a non-protein thiol, can chemically react with O_2_^−^, ^−^OH, and H_2_O_2_, acting as a powerful radical scavenger [94]. In addition, during normal and stressful conditions, GSH acts as a substrate for dehydroascorbate reductase to synthesize AsA [97]. GSH’s reducing capability also influences protein synthesis, enzymatic control, and the expression of genes that respond to stress [94,95]. The ratio of reduced GSH to oxidized glutathione (GSSG) in this context is an important indicator of the redox status of the cell. According to Gill and Tuteja [95], glutathione reductase (GR; EC 1.6.4.2) increases GSH biosynthesis and/or GSSG degradation or, alternatively, their long-distance transport to achieve enhanced levels of GSH compared to GSSG. According to Castro, Lima-Melo, Carvalho, Feitosa, Lima Neto, Caverzan, Margis-Pinheiro, and Silveira [96], specific enzymes called glutamyl cysteine ligase and glutathione synthetase synthesize glutathione in the cytosol and chloroplasts. However, glutathione has also been found in vacuoles, the endoplasmic reticulum, and mitochondria [96]. 

#### 5.2.3. Carotenoids

As one of the most prevalent naturally occurring pigments, carotenoids are produced by both photosynthetic and non-photosynthetic organisms. They belong to a class of lipophilic chemicals with over 700 species [98]. They can be divided into two groups: the carotenes, which are oxygen-free carotenoids (e.g., β-carotene and lycopene), and the xanthophylls, which are their oxygen-containing counterparts (e.g., lutein and zeaxanthin) [99]. These low-molecular-weight metabolites can prevent the synthesis of ^1^O_2_ in photosynthetic organs by quenching excited chlorophyll and triplet sensitizer, safeguarding the photosynthetic apparatus, and lowering lipid peroxidation [96]. According to Havaux [99], because β-carotene is close to the main location of ^1^O_2_ production in chloroplasts, its oxidation can be thought of as an early event during photo stress. As a result, metabolites from β-carotene oxidation may serve as the primary sensors of light stress in plants. Carotenoids and their byproducts are essential for the construction of photosystems as well as for the regulation of developmental processes by directly regulating the production of two plant hormones, strigolactones and abscisic acid (ABA), since carotenoids serve as their precursors. Carotenoids also play a crucial role in the assembly of antenna complexes as accessory pigments responsible for absorbing light at 400 and 550 nm [93].

#### 5.2.4. α-Tocopherols 

A unique quantity and placement of methyl groups in the 2-methyl-6-cromanol ring distinguish the four isomers of tocopherols (α-, β-, γ-, and δ-tocopherol). However, the most prevalent isomer is α-tocopherol, having the highest AOX activity [100]. Tocopherols are located in plastids, plastoglobuli, and in close proximity to the envelope and thylakoid membranes. Since it may directly interact with ^1^O_2_, ^−^OH, and certain lipid radicals produced by the oxidation of polyunsaturated fatty acids, α-tocopherol is especially active in the thylakoid membranes, where it can stop lipid peroxidation. Through an energy transfer process, α-tocopherol can neutralize ^1^O_2_, resulting in the synthesis of various quinones and epoxides. One of these compounds, α-tocopherol quinone, demonstrates antioxidant characteristics similar to those of α-tocopherol and appears to be involved in PSII energy dissipation [101]. 

Because quinones and other oxidized derivatives cannot be converted back into α-tocopherol, the AOX characteristics may be compromised. Contrarily, when α-tocopherol reacts with alkoxy or peroxyl radicals, it creates tocopheroxy radicals, which allow the intervention of AsA, GSH, and coenzyme Q to regenerate α-tocopherol [102]. α-tocopherol preserves the membrane’s integrity in chloroplasts and makes these structures stiffer, which affects their fluidity and permeability for ions and small molecules [101]. Because of the role of α-tocopherol in membrane stability, its contribution to chloroplast redox homeostasis, and its ability to control the concentration of some phytohormones, such as jasmonic acid, it is assumed that α-tocopherol may interact with key players in signal transduction pathways, indicating that tocopherol has functions beyond its antioxidant activity [96].

#### 5.2.5. Phenolic Compounds

Flavonoids, tannins, hydroxycinnamate esters, lignin, and other secondary metabolites, collectively referred to as phenols, are found in plant tissue [103]. The ability of phenols to act as antioxidants is due to the fact that they have an aromatic ring structure with ^−^OH or ^−^OCH_3_ substituents, which is excellent for trapping free radicals. Phenolic compounds directly absorb ^1^O_2_ and prevent peroxidation of the lipid by seizing lipid alkoxy radicals [94]. The capacity of phenols to affect the kinetics of peroxidation by changing the lipid package and decreasing membrane fluidity is another mechanism correlated with their antioxidant effects. These modifications may restrict free radicals’ ability to diffuse and may lessen the peroxidation process. Additionally, it has been demonstrated that phenolic compounds may play a role in the H_2_O_2_ capture cascade [84]. 

Non-enzymatic antioxidants, including AsA, GSH, α-tocopherol, phenolic compounds, flavonoids, alkaloids, and non-protein amino acids, cooperate with antioxidant enzymes including SOD, CAT, POX, polyphenol oxidase (PPO), APX, MDHAR, DHAR, GR, GPX, GST, TRX, and PRX in order to control the rapid production of ROS [104]. 

The efficiency and behavior of the non-enzymatic antioxidant system under stress depend on diverse factors, such as the type of stress, time of exposure and its intensity, plant species and their genotypes, organ or tissue, among others. Therefore, distinct responses and outcomes from the non-enzymatic antioxidant system have been largely reported in plants under exposure to different biotic and abiotic stresses [93].

## 6. RNS Production in Plants

**Figure 3:**
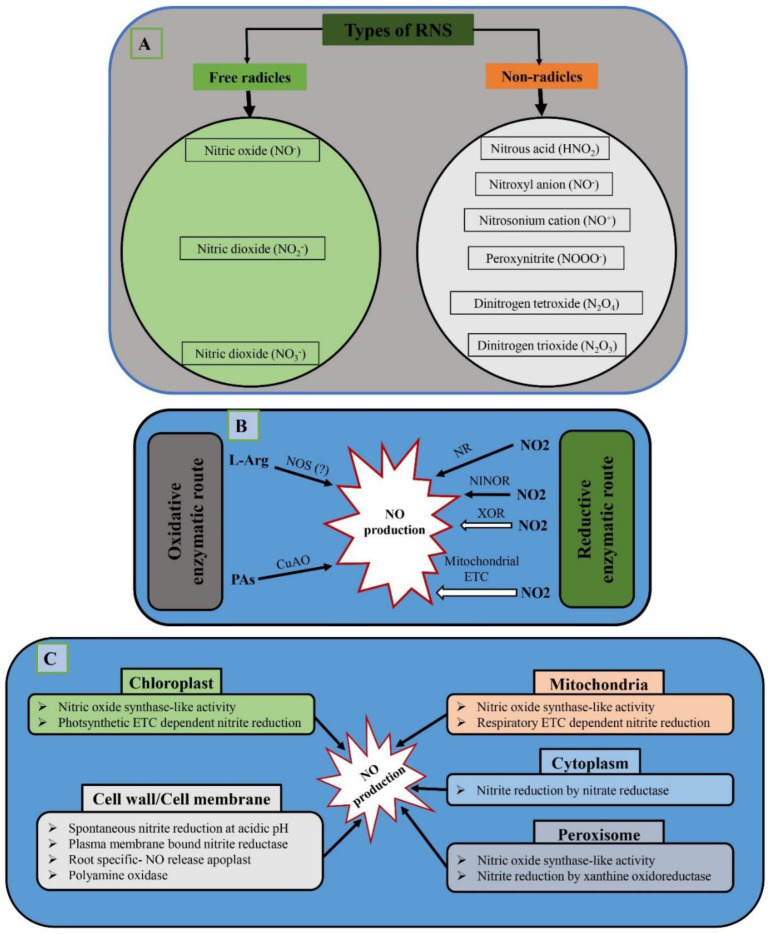
(**A**) All types of reactive nitrogen species (ROS) in plants, (**B**) oxidative and reductive pathways, (**C**) sites of NO production.

RNS is a collective term that includes radicals such as a nitric oxide (NO^−^) and nitric dioxide (NO_2_^−^) and non-radicals such as nitrous acid (HNO_2_) and dinitrogen tetroxide (N_2_O_4_) [105]. All types of RNS are shown in Figure 3A. Among the above, the most important RNS is nitrogen oxide, which acts as a significant signaling molecule in all living organisms and was proclaimed the “molecule of the year” in 1992 by the journal *Science*. As compared to ROS, the mechanism of NO production in plant cells is not yet fully explored, which is one of the main obstacles in the investigation of NO as a signaling molecule. In animals, NO is produced mostly by nitric oxide synthase (NOS). Furthermore, though there are many reports of the role of nitric oxide synthase (NOS) in the synthesis of NO in the extracts of different plant species, its occurrence in higher plants has not been explored yet [106]. On the other hand, it has been found in the unicellular alga *Ostreococcus tauri* [107]. The proposed pathways for NO production are oxidative and reductive. The substrates in the oxidative pathway for NO production are arginine, hydroxylamine, and polyamines [108]. The reductive pathway includes the action of xanthine oxidoreductase (XOR) in the peroxisomes and nitrite:NO-reductase (NiNOR) reductase attached to the membrane [15]. In plants, an important source of NO production is cytosolic enzyme nitrate reductase (NR). It has been suggested that NR plays a vital role in NO production during bacterial defense, in the presence of other pathogens, under drought stress, during cold acclimation, in stomatal regulation, in diminishing the symptoms related to iron deficiency, and in the process related with the growth of the roots [15]. The previous results also show that under normal conditions, NR prefers to convert nitrate to nitrite, and it increases NO production under certain conditions, such as anaerobic conditions, or at a higher level of nitrate [109]. The pathways of NO production are shown in Figure 3B. The cell organelles involved in the production of NO are the chloroplast, mitochondria, cytoplasm, cell wall, cell membrane, and peroxisome [15]. Furthermore, S-nitrosothioles such as S-nitrosoglutathione (GSNO) also play a significant role in the increased NO concentration inside plants, and may act as an NO donor [110]. The sites of NO production are shown in Figure 3C.

## 7. Role of RNS in Plant–Microbe Interactions

The mechanisms that activate a variety of defenses, including the cross-linking of cell wall proteins, the production of ROS and RNS, localized PCD, and the activation of pathogenesis-related (*PR*) genes both at local and systemic sites, determine a plant’s resistance to disease [8]. This system of defenses involves unique plant receptors that can detect various signals given out by the pathogens and guards the whole plant tissues from a wide variety of infections [37]. NO is produced in response to potential aggressors such as viruses and microbial pathogens during biotic stress and is involved in a variety of stress responses. These reactions range from the control of defense genes to the synthesis of hormones and the emergence of the hypersensitive response (HR) [72,111]. The role of NO in PTI, ETI, and symbiotic association and the role of GSNOR and NR in its homeostasis during plant immunity have been discussed in the following sections in detail. 

### 7.1. Role of NO in PTI, ETI, and Symbiotic Association in Plants

It is suggested that plant innate immunity is a two-tiered immune system with both PTI and ETI. Furthermore, SAR is also an important defense response of the plants against pathogenic bacteria. In fact, it has been demonstrated that plants produce NO quickly after being challenged by biotrophic and necrotrophic diseases, and it is regarded as a key defense activator [56]. It is widely acknowledged today that NO generated in response to MAMPs/PAMPs and effector molecules from the pathogens has a significant impact on signaling pathways [6]. 

As a chemical messenger, after the recognition of pathogens via the interaction between MAMPs/PAMPs and PRRs, NO significantly enhances the expression of pathogenesis-related (PR) genes, including *PR1* and *PR2,* and significantly reduces pathogenic growth. It reveals that NO positively regulates plants’ basal defense/PTI. For instance, after the inoculation of the virulent bacteria DC3000, the expression *AtPR1* and *AtPR2* was more significantly enhanced in wild-type Col-0 than in *atgsnor1-3* and NO-induced mutant line *atill6*. Furthermore, symptomless development of severe diseases and increased pathogenic growth was observed in *atgsnor1-3* and the NO-induced mutant line *atill6* as compared to Col-0 WT [8]. Feechan, Kwon, Yun, Wang, Pallas, and Loake [74] also stated that S-nitrosoglutathione reductase (GSNOR) is required for basal and *R*-gene-mediated resistance. NO accumulation is crucial to increase the basal defense system of the Arabidopsis against *Phytophthora parasitica* infection [112]. The same author also suggested that the loss-of-function mutant line of GSNOR1 was deficient in SA accumulation and the signaling associated with it, which led to its susceptibility towards infection with *Phytophthora parasitica*. Furthermore, Shahid, Imran, Hussain, Khan, Lee, Mun, and Yun [72] also found that NO-induced *AtCL1* and *AtCL2* positively regulated plants’ basal defense with an increase in the transcript accumulation of *AtPR* genes and lower pathogenic growth. On the other hand, Nabi, Rolly, Tayade, Khan, Shahid, and Yun [37] and Khan, Imran, Shahid, Mun, Lee, Khan, Hussain, Lee, and Yun [56] observed that NO-induced *AtbZIP62*, *AtAO3*, and *AtNCED3* genes negatively regulated PTI resistance against virulent bacterial infection.

Similarly, NO also significantly contributes to ETI and *R*-gene-mediated resistance. Imran et al. [113] found that the NO-induced *AtHMAD1* gene positively regulated ETI and *R*-gene-mediated resistance. They observed higher pathogenic growth and electrolyte leakage and lower transcript accumulation of *AtPR* genes in *athmad1* and *atgsnor1-3* mutant lines as compared to Col-0 WT. These results show that GSNOR is required for the induction of ETI and *R*-gene-mediated resistance against a virulent bacterium (*avr*B). Khan, Nazar, Pande, Mun, Lee, Hussain, Lee, and Yun [8] found similar results, and they suggested that the NO-induced gene *AtILL6* positively regulated ETI and *R*-gene-mediated resistance against the infection of *avr*B. In contrast, the NO-induced *AtAO3* and *AtNCED3* genes from the ABA pathway negatively regulated plants’ ETI with decreased transcript accumulation of *AtPR* genes and higher pathogenic growth in Col-0 WT than the *atbzip62*, *atao3*, and *atnced3* mutant lines. However, these NO-induced genes positively regulated the defense system of *A. thaliana* plants against drought stress [37,56].

In the past two decades, numerous studies have been conducted on the mechanisms driving plant–microbe interaction in the rhizosphere [17]. Interaction between plants and microbes takes place through a highly complicated communication network that uses cutting-edge technology on both sides to identify friends and enemies. The role of NO during recognition, root hair curling, the formation of infection threads, nodule development, and nodule senescence is suggested by studies on the legume–rhizobia symbiosis [5]. The interaction of plants and mycorrhizal fungus is also thought to involve a similar role for NO. It is interesting to note that there have been findings linking silicon to an increase in nodules, an improvement in nitrogen fixation, and a possible interaction between silicon and NO in mediating microbial communication underground [5]. 

### 7.2. S-Nitrosylation of NPR1, SAB3, PAL, and CHS Proteins

According to Durrant and Dong [114], SA is a key signaling molecule in plant immunity. The non-expresser of pathogenesis-related protein 1 (NPR1) is a crucial transcription factor (TF) in the SA-mediated pathway. According to Mou, et al. [115], NPR1 activity is redox-sensitive, and its oxidized oligomeric form is found in the cytoplasm. When a pathogen infects a cell, a rise in SA causes Cys residues to be reduced, which causes NPR1 to monomerize and quickly go to the nucleus. Expression of the defense-related genes is enhanced in the nucleus by NPR1’s interaction with co-transcriptional factors of the TGA family [114]. Furthermore, NPR1 is also regulated by S-nitrosation, which is controlled by NO or GSNO concentrations. S-nitrosation induces the oligomerization of NPR1 genes and thus helps to regulate the level of NPR1 in the cytoplasm [116]. Other researchers, however, have demonstrated that NO facilitates NPR1 translocation to the nucleus, where it interacts with S-nitrosylated TGA1, increasing TGA1’s DNA-binding activity [117]. In considering these contrasting results, it has been proposed that S-nitrosation-mediated oligomerization might not have an inhibitory effect on NPR1 activity but may act as an earlier step to monomer accumulation, supporting the proposition of a positive effect of NO or GSNO on the resistance of plants to disease. Furthermore, Lindermayr, Sell, Muller, Leister, and Durnera [117] suggest that a secondary action inducing S-nitrosation of NPR1 might happen once this protein is already in the nucleus. 

Additionally, the SA-binding protein SAB3, which acts as a positive regulator of plant immunity, is also a target for S-nitrosation. Due to S-nitrosation, the post-translated modified SAB3 loses its SA-binding potential and strongly reduces its carbonic anhydrase activity, which is necessary for immune signaling [118]. Besides this, NO also induces the expression of defense-related genes, such as encoding phenylalanine ammonia-lyase (PAL) and chalcone synthase (CHS), two important enzymes of the phenylpropanoid pathway required for the production of flavonoids with antimicrobial activity. The research, which was conducted on different plants, including potato, soybean, and wheat plants, also supported the key role of NO in the synthesis of antimicrobial compounds in plant–pathogen interaction [119].

### 7.3. Role of GSNOR and NR in NO Homeostasis and Plant Immunity

In response to pathogenic bacteria, NO is produced by the action of NR. This was confirmed by using the loss-of-function of NR *atnia1–atnia2* double mutant, which showed reduced NO emissions and an impaired HR when inoculated with avirulent bacteria or *Sclerotinia sclerotiorum* [120,121]. The amino acid levels in *atnia1–atnia2* mutants are also decreased; treatments with glutamine were able to restore the WT amino acid levels but not the resistance to avirulent bacteria [122], disproving the possibility that the plant susceptibility was caused by problems with nitrogen metabolism. The hypothesis that NR is solely required to generate the nitrite required for NO synthesis is supported by the fact that the penetration of *atnia1–atnia2* leaves by nitrite results in enhanced NO emissions and activation of the HR in pathogen-challenged plants [120,123]. Furthermore, NR-dependent NO production is also observed in response to abiotic stress conditions and in nitrogen-fixing nodules.

According to [74,124], plants with null or reduced expression of GSNOR have higher levels of total S-nitrosothiols (SNO), whereas plants with GSNOR overexpression have lower levels of SNO. The modulation of intracellular SNO levels by the GSNOR enzyme has significant ramifications for plant immunology. According to Rustérucci, Espunya, Díaz, Chabannes, and Martínez [124], who used an antisense approach, plants with decreased GSNOR activity (50%) displayed improved basal resistance and improved induced SAR, whereas plants with GSNOR overexpression displayed increased susceptibility to pathogens in comparison to WT plants. Surprisingly, other researchers demonstrated that *AtGSNOR1* null mutants had reduced basal and pathogen-induced (gene-for-gene) resistance [74]. A striking difference between these two types of mutants was that the content of SA, which is similarly essential for plant immunity [124,125], was not altered in the antisense plants but was significantly decreased in GSNOR-null mutants. Additionally, exogenous SA had no effect on GSNOR-null mutants [74,126]. If the intricate control of NPR1 is taken into account, the seemingly conflicting results obtained with null [74] and antisense [124] mutants might be reconciled. As a result, Espunya, De Michele, Gómez-Cadenas, and Martínez [125] hypothesized that decreased levels of GSNOR activity in the antisense plants might favor the existence of the proper ratio of S-nitrosylated/NPR1/TGA1 forms, which would have a positive impact on plant defense. GSNOR knockout mutants may completely hinder activation of the NPR1/TGA1 signaling pathway by their inability to prevent the overaccumulation of GSNO.

**Figure 4:**
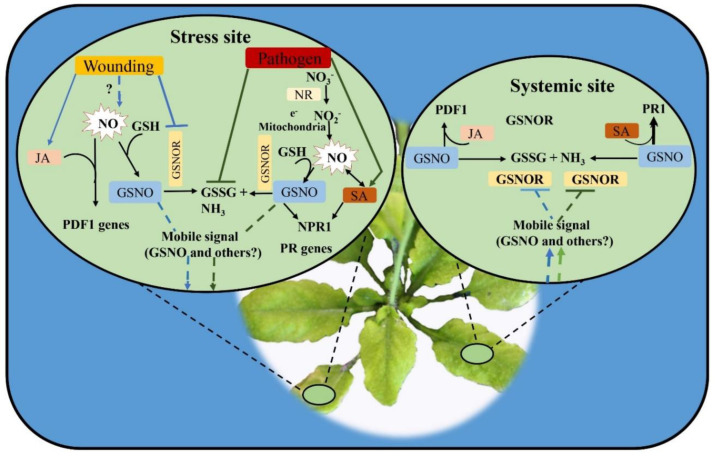
Proposed model of action for GSNOR and NR in NO homeostasis and plant immunity. After the exposure to the pathogen, activation of NR at the local site leads to accumulation of NO_2_^−^, which is reduced to NO by the mitochondrial electron transport system. The new production of NO rapidly increases the concentration of GSNO and other nitrosothiols; moreover, the transcriptional inhibition of GSNOR also contributes to maintaining the enhanced GSNO pool.

Additionally, it has been revealed that GSNOR may play a significant role in regulating systemic defense responses during pathogenic and wound stress (Figure 4). According to Díaz, et al. [127], wounding, SA, and JA both locally and systemically affect the transcription of the gene GSNOR. Espunya, De Michele, Gómez-Cadenas, and Martínez [125] demonstrated that in wounded Arabidopsis leaves, GSNO levels grew quickly and uniformly, whereas in systemic leaves, GSNO was initially found in vascular tissues before spreading over the parenchyma. These findings imply that GSNO participates in the movement of the wound mobile signal across the vascular tissue. Furthermore, the alternative JA-independent wound-signaling pathway does not use GSNO, whereas JA-dependent wound responses need GSNO accumulation to be activated. In response to diverse abiotic stimuli, GSNOR also modifies SNO levels, which is crucial for resistance and acclimatization [128]. 

## 8. Cross-Talk of ROS and RNS

In response to biotic and abiotic stress conditions, both ROS and RNS are produced in significant amounts and cause oxidative stress. However, at optimum concentrations, they act as chemical messengers to regulate growth and development and induce a defense response against these stressors. In plants, the production of RNS and ROS is a common requirement for cells to go through PCD. These tiny molecules can operate either alone or cooperatively [129]. Although the precise role of RNS and ROS in the process of cell death is still unclear, the mounting evidence suggests that RNS and ROS interact significantly. ROS and NO can control one another’s production. NO can influence the generation of ROS during the HR by S-nitrosylating the NADPH oxidase AtRBOHD [126]. However, in the rice *noe1* mutant, the loss of OsNOE1/OsCATC function causes the accumulation of H_2_O_2_ to stimulate NO generation by increasing nitrate reductase expression. Nitrate reductase is also crucial for the H_2_O_2_-induced death of leaf cells through the S-nitrosylation of GAPDH and thioredoxin [130,131]. The HR, which is characterized by fast cell death around infection sites, is a well-researched plant PCD. Cross-talk between NO and H_2_O_2_ is a key aspect of these tiny molecules’ activity. Additionally, RNS and ROS are crucial in controlling the activity of target proteins. The HR procedure is under the jurisdiction of both RNS and ROS. The balance between intracellular NO and ROS levels is one of the major factors affecting the HR [132,133]. When a pathogen is detected, NO builds up concurrently with an oxidative burst, which includes a phasic synthesis of apoplastic ROS at the location of the attempted invasion [134]. NO and H_2_O_2_ are believed to work together in this context to cause HR cell death. For example, each of them could trigger the release of cytochrome C from mitochondria and impact the caspase-like signaling cascade that results in the HR [129]. Mitogen-activated protein kinases (MAPKs) and phosphatases are some important defensive signaling cascade elements known to be impacted by ROS and NO activity. Therefore, altering the core MAPK cascade may result in the combination of the H_2_O_2_ and NO signaling pathways, which are triggered in response to pathogen infection. Upon xylanase sensing in tomato cell suspensions, cells activate a protein kinase pathway necessary for NO synthesis as well as S-nitrosylation-dependent mechanisms that are involved in downstream signaling, resulting in the production of polyamine and ROS [135]. It is interesting to note that numerous proteins are both NO and H_2_O_2_ targets. For instance, H_2_O_2_ directly targets GAPDH, which is involved in mediating ROS signaling in plants, and NO-mediated S-nitrosylation targets it as well, which reduces glyceraldehyde-3-phosphate dehydrogenase’s (GAPDH) activity [136]. Additionally, H_2_O_2_ inactivates methionine adenosyl transferase (MAT) in mammals by oxidizing a Cys residue in a reversible and covalent manner. NO also targets the same Cys residue, inducing comparable enzyme inactivation [137]. Further, PrxII E not only reduces H_2_O_2_ and alkyl hydroperoxides [129], but also functions in detoxifying peroxynitrite. During the defensive reaction, PrxII E undergoes S-nitrosylation, which controls the antioxidant function of this important enzyme and may be related to the HR [129]. The ability of ozone (O_3_) to cause HR-like cell death makes it a powerful instrument for inducing ROS-activated responses. This suggests that NO is a key signaling molecule in response to O_3_ exposure because NO accumulation occurred prior to buildup of ET, JA, SA, and leaf damage [138,139]. Contrary to its intended role in the HR, NO can also scavenge H_2_O_2_ and, in some situations, shield plant cells from harm [140,141]. Both wounding- or JA-induced expression of defense genes and wounding-induced H_2_O_2_ production are impacted by NO donors [142]. GSNOR1 in Arabidopsis is a significant regulator that indirectly regulates the levels of protein S-nitrosylation on a global scale. Increased levels of total cellular NO and SNO were caused by loss-of-function mutations in GSNOR1, which also impaired resistance (R) gene-mediated defense and hindered basal defensive mechanisms [74,143]. Additionally, the mutant *atgsnor1-3* showed altered thermotolerance and resistance to paraquat (1,1′-dimethyl-4,4′-bipyridinium dichloride), a chemical that causes the generation of superoxide and H_2_O_2_ in the leaves of the WT [129]. These findings are supported by the fact that WT plants treated with an NO donor showed paraquat resistance [144]. These investigations demonstrated that the Arabidopsis *GSNOR1*/*HOT5*/*PAR-2* gene controls cell death by acting downstream of superoxide, in addition to regulating SA signaling and thermotolerance. It is interesting to note that the HR was potentiated in *atgsnor1-3* plants by higher levels of SNOs even in the absence of apoplastic ROS production and the cell death agonist SA. Unexpectedly, NO S-nitrosylation of the NADPH oxidase, AtRBOHD, at Cys890, reduces its capacity to produce ROS. Additionally, this cysteine is preferentially S-nitrosylated in both human and fly NADPH oxidases, indicating that this mechanism may control immunological responses in both plants and animals [126]. In order to further manage the onset of cell death processes, NO may therefore regulate the formation of ROS through protein S-nitrosylation. Together, these results shed light on the processes underlying ROS and RNS function in plants and reveal that the ROS/RNS pathway in plant PCD is extremely complicated is at least partially regulated by cross-talk with a number of phytohormone signaling networks [129].

Other types of plant cell death are also described in some papers as involving NO and ROS interplay. Aleurone layer PCD caused by gibberellin (GA) in barley is mediated by ROS, while NO is a protective antioxidant [129]. While delaying this PCD process, NO donors do not impair the overall metabolism or the GA-induced production and release of alpha-amylase. In aleurone layers treated with GA, the levels of CAT and SOD are significantly decreased. In the presence of NO donors, treatment with GA slows the depletion of CAT and SOD. Consequently, NO may act as an endogenous PCD modulator in barley aleurone cells [140]. NO may potentially support PCD brought on by ROS. Self-incompatibility (SI) causes relatively quick and temporary increases in ROS and NO during pollen–pistil interactions. Since ROS/NO scavengers reduced the development of SI-induced actin punctate foci and the activation of a DEVDase/caspase-3-like activity, both of these effects were reduced [145]. Sphinganine, or dihydrosphingosine (d18:0, DHS), induces calcium-dependent PCD and causes H_2_O_2_ generation via activating NADPH oxidase in tobacco BY-2 cells (s). It also encourages the synthesis of NO, which is necessary to trigger cell death [146]. By encouraging MPK6-mediated caspase-3-like activation, NO accumulated in Cd-induced PCD and aided Cd-induced PCD in Arabidopsis [147]. Therefore, the many functions of RNS in PCD and their interactions with ROS rely on the type of plant, the environment it grows in, and its redox state.

## 9. Role of ROS and RNS in Protein Modifications

Protein oxidation is described as the covalent alteration, a significant class of post-translational modification, of a protein brought on either directly by interactions with ROS or indirectly by conjugation with breakdown products of fatty acid peroxidation. Nitrosylation, carbonylation, disulfide bond formation, and glutathionylation are all examples of direct modifications that affect a protein’s activity [94]. The amino acids arginine, histidine, lysine, proline, threonine, and tryptophan are preferentially targeted by indirect modifications of proteins, which increases the vulnerability of proteins to proteolytic disintegration [95]. Since thiol groups and S-containing amino acids, such as Met and Cys, are more likely to be attacked by ROS and are particularly susceptible to oxidation, they are the most often changed amino acids [94]. Cys residues can have a H atom removed by active oxygen, creating a thiyl radical that then bonds with another thiyl radical to create a disulfide bridge [148]. Sulfenic acid, sulfinic acid, and sulfonic acid derivatives can also be produced by the oxidation of protein Cys thiol groups [149]. However, Met can also be subjected to ROS-mediated oxidation, just as Cys. Protein Met residues undergo oxidation to form methionine-S- and methionine-R-sulfoxides [149]. Methionine sulfoxide reductase, a group of cytosolic and plastidic enzymes involved in reducing oxidative damage, quickly reduces the oxidized Met residues back to Met [93]. However, superoxide radical irreversibly inactivates enzymes that contain Fe-S centers, resulting in enzyme inactivation [150].

Proteins that have been permanently inactivated cannot be restored; instead, they must be identified and removed by cellular proteolytic processes [94]. These removal and degradation processes are crucial for the preservation of cellular metabolism. By preparing for ubiquitination and becoming a target for proteasome destruction, it has been proposed that oxidized proteins are better substrates for proteolytic digestion [151]. The most frequent oxidative protein modification, carbonylation, is an irreversible alteration of proteins that has a detrimental effect on the structure and functionality of proteins that are involved in channels, enzymes, and receptors [150]. The cytoplasm, chloroplasts, peroxisomes, nucleus, and mitochondria of plant cells have all been reported to have carbonylated proteins. According to Bartoli, et al. [152], wheat leaves had a larger concentration of carbonylated proteins per mg of protein in the mitochondria than in the chloroplasts and peroxisomes, which may indicate that mitochondrial proteins are more vulnerable to oxidative damage. The carbonylation of proteins is caused by both biotic and abiotic stresses, such as heat, salinity, drought, and heavy metals. However, the degree of carbonylation is correlated with the stressor factor’s severity and exposure period [153].

NO mediates its redox functions primarily through S-nitrosation/nitrosylation, which is the addition of an NO moiety to a Cys sulfhydryl/thiol to form an SNO. It has been demonstrated that this redox-based alteration controls plants’ immunity, development, and responses to the environment. According to newly available data, NO orchestrates some of these processes by controlling the use of various PTMs [154]. The small ubiquitin-like modifier (SUMO), which is attached covalently to target proteins, is emerging as a crucial regulator of eukaryotic immunological activity. In plants, it has been suggested that SUMO1/2-dependent mechanisms regulate the activation of host immunity [155]. S-nitrosation has recently been shown to have a significant role in the regulation of SUMOylation [156]. Increasing NO levels were shown to cause S-nitrosation of the Arabidopsis SUMO E2 enzyme SCE1 at Cys139 after a pathogen-triggered nitrosative burst. Therefore, the accumulation of SUMO1/2 conjugates, impaired immunological responses, and increased vulnerability to pathogens were caused by the mutation of Cys139 [156]. Taking these results together, it was determined that S-nitrosation of SCE1 at Cys139 allows NO bioactivity to increase immunological activation by reducing SUMO1/2-mediated repression. 

According to newly available information, NO plays a significant role in regulating phosphorylation-dependent signaling cascades. NO buildup has been shown to activate protein kinases (PKs) and cause the phosphorylation of several proteins involved in a variety of cellular activities [157]. Ca^2+-^dependent PKs (CDPKs), sucrose nonfermenting 1-related PKs (SnRKs), mitogen-activated PKs (MAPKs), and phosphoinositide-dependent PKs are examples of NO-dependent PKs (PDKs). However, it is still unknown how NO modifies the activity of these target PKs. By releasing cytosolic free Ca^2+^, NO is hypothesized to indirectly mediate the activation of MAPKs and CDPKs [158]. But it is still unknown what subtle mechanisms underlie this process. SnRKs, MAPKs, and CDPKs have not been reported to undergo direct S-nitrosation [159]. However, it was shown that S-nitrosation of a crucial catalytic Cys residue decreased the action of tomato cell death regulator PDK1. Additionally, preliminary results suggest that tyrosine nitration may be used to regulate the activity of MAPKs [160]. In fact, the dual phosphorylation of the Thr-X-Tyr motif in the activation loop by MAPK kinases (MAPKKs) causes MAPKs to become active. Consequently, it is tempting to hypothesize that nitration of the Tyr residue inside the activation loop may prevent it from being phosphorylated by MAPKKs and, as a result, negatively affect MAPK activity. Finally, NO may affect phosphorylated PK and phosphorylated proteins more generally by redox-regulating protein phosphatases (PPs). Major phosphatases, particularly tyrosine phosphatases, are impacted by this process, which is well known in mammals [161]. 

In eukaryotic species, the chromatin structure is extremely dynamic and changes during growth and development as well as in response to environmental cues. Histone protein modification triggers chromatin remodeling to regulate transcription, replication, recombination, and repair [162]. These processes depend on the modification of histone acetylation or methylation, which is catalyzed by enzymes called histone acetyltransferases/histone deacetylases (HDAs) and methyltransferases/demethylases, respectively [154]. NO has recently been shown to influence histone acetylation by inhibiting histone deacetylase (HDA) complexes [163]. NO-regulated histone acetylation of genes essential to immunity, abiotic stress, and chloroplast function was discovered via genome-wide NO-dependent H3K9/14ac profiling in Arabidopsis seedlings, indicating that NO bioactivity may regulate gene expression by modulating chromatin structure [163]. Furthermore, it has been demonstrated that NO buildup causes worldwide DNA hypomethylation, which changes the expression of chromatin-remodeling enzymes [164]. This suggests that NO indirectly affects plant chromatin methylation processes. The current facts collectively imply that NO bioactivity might play significant functions in the nucleus, but further research into the molecular specifics is still needed.

## 10. Conclusions and Future Prospects

It is widely understood that ROS and RNS play a key role as signaling molecules in plant growth and development under normal, biotic, and abiotic stress conditions. However, the beginning of ROS and RNS signaling, the detection and response mechanisms, and how the balance between the synthesis and removal of these species is managed are all areas in which much remains to be discovered. Although recent approaches such as RNA-seq and proteomics have enabled discoveries of new TFs and protein–protein interaction that support cellular ROS and RNS signaling in plants, fundamental knowledge about ROS and RNS synthesis and their activities inside the plant is still needed. There is still little information about the role of ROS and RNS in the initiation of the signaling network, the mechanism required for the perception and specificity of the general signals, and the regulation between the production and elimination of ROS and RNS in plants. However, advanced microscopy techniques and in vivo optical manipulation of intracellular structures are providing new windows into the synthesis and activities of ROS and RNS inside the plant. Understanding how the various metabolic pathways regulate the production and elimination of both ROS and RNS, the post-translational modifications mediated by ROS and RNS, and their signaling role in different physiological and metabolic processes and responses to biotic and abiotic stresses in plants should constitute the major objectives of ROS and RNS research going forward. 

In this review, we discussed the vital role of ROS and RNS as key signaling molecules in plant–microbe interactions and the antioxidant system as a regulator in the synthesis and elimination of these species. Both ROS and RNS, as significant signaling molecules, have gained great fame amongst plant scientists in the last few decades, opening new windows into many aspects of plant physiology and metabolism, both under normal and harsh environmental conditions. Our understanding of the role of both ROS and RNS in the defense mechanism as signaling molecules is still relatively poor, and we still need some basic information about the enzymatic sources involved in the production of ROS and RNS in plant–microbe interactions. The attempts of many scientists to unveil the signaling role and sources of synthesis of ROS and RNS during plant–microbe interactions have been quite successful, and various reports from previous research indicate that antioxidants, NR, and GSNOR are important regulators of these processes. Hence, recognition and a description of the role of ROS and RNS in synthesizing enzymes during plant–microbe interactions as well as that of genes contributing to ROS and RNS signaling during the HR is necessary. To achieve these goals, genetic screening is being investigated as an interesting approach. Additionally, a functional genomics study is necessary to understand the mechanism and role of ROS and RNS in plant–microbe interactions. 

## Figures and Tables

**Table 1:** Roles of ROS and RNS in plant–microbe interaction.

Plant	ROS/RNS	Effects	References
Sand pear	H_2_O_2_	HR-mediated cell death	[80]
Arabidopsis	H_2_O_2_	Programmed cell death (PCD)	[81]
*Nicotiana benthamiana*	SOA and H_2_O_2_	Replication of two unrelated RNA viruses	[63]
Arabidopsis	ROS- and JA-dependent process	Lignin biosynthesis	[64]
Arabidopsis	Oxidative burst	PTI	[65]
Arabidopsis	RBOHD	Plant immunity	[66]
Tobacco	H_2_O_2_	PCD and HR	[67,68]
*Medicago truncatula*	ROS	Symbiosis	[69]
*Phaseolus vulgaris*	ROS	Symbiosis	[70]
*Castanea sativa*	ROS	Symbiosis	[71]
Arabidopsis	NO	Plant immunity	[8,37,72]
Arabidopsis	NO	Plant immunity, growth, and many more	[5,6,57]
Arabidopsis	NO	Plant immunity	[37]
Rice	NO	Resistance to viral infection	[73]
Tobaco	NO	Resistance against tobacco mosaic virus	[82]
Wheat	SNO	Resistance against powdery mildew invasion	[74]
*Medicago truncatula*	NO	Symbiosis	[75]
*Lotus japonicus*	NO	Symbiosis	[76]
*Medicago truncatula*	NO	Symbiosis	[77]
Legumes	NO	Symbiosis	[78]
